# EFFICACY OF *CURCUMA LONGA* IN THE TREATMENT OF DIVERSION COLITIS IN RATS

**DOI:** 10.1590/0102-672020190001e1456

**Published:** 2019-12-09

**Authors:** Arthur Medeiros LIMA, Carlos Eduardo Costa NASCIMENTO, Carlos Henrique Marques dos SANTOS, Doroty Mesquita DOURADO, Gabriel Elias Cardoso SIQUEIRA, Giovana Maria RIGO, Lauren Umpierre BERNARDI, Paulo Otávio Souza LEONEL, Rosemary MATIAS, Vitor Caldas FERREIRA, Vitor Cruz Rosa Pires de SOUZA

**Affiliations:** 1 University Anhanguera-Uniderp, Campo Grande, MS, Brazil; 2 Federal University of Mato Grosso do Sul, Campo Grande, MS, Brazil

**Keywords:** Curcuma, *zingibereceae*, Colite, Anti-inflamatórios, Ratos, Colostomia, Curcuma, *zingibereceae*, Colite, Anti-inflamatórios, Ratos, Colostomia

## Abstract

**Background:**

Diversion colitis is still very common in our country, since the stoma creation is a common practice especially in situations of trauma. needing treatment for this condition.

**Aim:**

To evaluate the therapeutic effect of rectal infusion of *Curcuma longa* (turmeric) in the excluded intestinal segment of rats.

**Method:**

Eighteen Wistar rats were used and submitted to colostomy: control group (n=8) under rectal saline infusion and group CL, receiving intra-rectal infusion of *Curcuma longa* extract (n=10). After 21 days of treatment they were submitted to euthanasia; the intestinal segment excluded from intestinal transit was resected and sent to histopathological evaluation, classifying the degree of inflammation and of vascular congestion.

**Results:**

The average of inflammation was 2.7 in the control group vs. 2.6 in the CL group (p=0.3125), while the mean vascular congestion was 2.3 and 2.1, respectively, in the control and CL groups (p=0.1642).

**Conclusion:**

Intra-rectal infusion of *Curcuma longa* extract was not able to minimize the inflammatory process or vascular congestion in the diversion colitis of rats subjected to colostomy.

## RESUMO

**Racional:**

A colite de exclusão é ainda muito frequente em nosso meio, uma vez que a confecção de estomas é prática comum especialmente em situações de trauma. Em consequência, há necessidade de tratamento para a colite de desuso.

**Objetivo:**

Avaliar o efeito terapêutico da infusão retal de *Curcuma longa* (açafrão-da-terra) no segmento intestinal excluso de ratos.

**Método:**

Utilizou-se 18 ratos Wistar submetidos à colostomiae distribuídos em dois grupos: grupo controle, recebendo infusão intrarretal de solução salina (n=8) e grupo CL, recebendo infusão intrarretal de extrato de *Curcuma longa* (n=10). Após 21 dias de tratamento foram submetidos a eutanásia; o segmento intestinal excluso de trânsito intestinal foi ressecado e submetido a estudo histopatológico classificando-se os graus de inflamação e de congestão vascular.

**Resultados:**

Verificou-se média de inflamação 2,7 no grupo controle vs. 2,6 no grupo CL (p=0,3125), enquanto as médias de congestão vascular foram 2,3 e 2,1, respectivamente, nos grupos controle e CL (p=0,1642).

**Conclusão:**

A infusão intrarretal do extrato de *Curcuma longa* não foi capaz de minimizar o processo inflamatório ou a congestão vascular na colite de exclusão de ratos submetidos a colostomia.

## INTRODUCTION

Is still common to perform intestinal stomas around the world, especially in emergency procedures such as trauma, intestinal obstructions, perforations and inflammatory bowel diseases. These diversions are mainly made on a temporary basis; however, may become definitive because of the clinical conditions of the patients. When temporary, an average of three months is estimated for intestinal reconstruction; this period may be extended for years due to multiple factors^4^ ^,^ ^17^.

The Hartmann’s procedure, when the sigmoid is externalized and the rectum excluded from the intestinal transit, corresponds to half of the intestinal transit deviations, making this type of stoma also the one with the greatest number of complications, especially in the long term. Thus, in cases where the deviation remains for more than six months, the number of complications increases, among them, diversion colitis^11^ ^,^ ^13^.

Diversion colitis can cause rectal pain in addition to mucopurulent secretion, causing discomfort to patients and may cause difficulties in the anastomosis at the time of bowel reconstruction when this is possible. The best therapy for this situation is the infusion of short-chain fatty acids enemas, since there would be direct nutrition of the intestinal cells through the lumen^1^. However, the biggest obstacle to such therapy is cost, which is quite high and unfeasible when treatment should be prolonged for long periods^2^ ^,^ ^5^.

Thus, the number of researches that seek a therapeutic option as effective as short-chain fatty acids and that present a lower cost without adverse effects is increasing, which theoretically would be achieved with herbal medicines, since many demonstrated an anti-inflammatory effect in other situations^14^ ^,^ ^18^.

Curcuma in medicine has been used for its anti-inflammatory and healing effect. Its bioactivity is due to curcuminoids, especially curcumin, which have several scientifically proven effects, such as reduction of inflammation in cases of arthritis, prevention of arteriosclerosis, respiratory and gastrointestinal disorders, hepatoprotective effects, skin conditions such as psoriasis and eczema, prevention of cancer and antioxidant capacity. The anti-inflammatory capacity, in part, is due to the inhibition of the synthesis of inflammatory prostaglandins^9^.

It was evidenced that the application of enemas containing oily extract of *Curcuma longa* was effective in preventing some inflammatory signs like epithelial loss and also the preservation of the regularity of the colonic lumen epithelium^8^.

Thus, there being evidences of the anti-inflammatory effect of *Curcuma longa* and due to the necessity of finding medication of great availability and low cost that has efficacy in the diversion colitis, the present research is justified, whose objective is to evaluate the anti-inflammatory effect of *Curcuma longa* in the intestinal segment excluded from rats submitted to terminal colostomy.

## METHODS

All the procedures were carried out according to the norms of the Brazilian College of Animal Experimentation (COBEA) and the study was approved by the Commission of Ethics in Animal Use of the University Anhanguera-Uniderp.

### Animals

Eighteen male Wistar rats weighing 250-300 g were obtained from the animal facility of the Federal University of Mato Grosso do Sul. The animals were assigned into two groups: 1) control group, n=8, subjected to infusion of saline solution for 21 days; 2) *Curcuma longa* group, n=10, subjected to colostomy and, after seven days, to the infusion of *Curcuma longa* enemas for 21 days*.*

### Surgical procedure

The animals were anesthetized by intraperitoneal injection of 2:1 soluction of ketamine hydrochloride 50 mg/kg and xylazine hydrochloride 20 mg/ml, respectively, at the dose of 0.1ml of the solution for each 100 g of weight. 

After the anesthesia was confirmed, the animals were fixed to the operative table with the four limbs abducted, abdominal trichotomy was performed, antissepsia with clorexidine 2%, and median laparotomy of approximately 4 cm was performed. A 6F probe previously inserted into the rectum marked the exact point of section to be performed similarly on all animals. The section was 6 cm proximally to the anus with cold knife scalpel, followed by closure of the distal stump with seromuscular continuous suture with polyglactin 910 5-0 thread. The proximal stump was externalized through the abdominal wall on the left and fixed to the skin by four points with polyglactin 910 5-0. The abdominal wall was closed by continuous suture with polyglactin 910 5-0 and the skin with 4-0 mononylon separated stitches.

### Preparation of *Curcuma longa* extract

The leaves were collected in their native vegetation areas of Campo Grande, Mato Grosso do Sul, Brazil. After drying, crushing and sieving were separately subjected to ethanol extraction, which was carried out in an ultrasonic bath (UNIDQUE, 1450) for 60 min and subjected to extraction by maceration until the drug was depleted. The solvents were evaporated (rotary evaporator at 45° C) and the crude aqueous extract remained in desiccator under reduced pressure for 6 h which was incorporated into the 0.9% saline solution. The established concentration was 200 mg/ml. For manipulation of the drug, the norms of good practices of manipulations of formulas were followed, with complete asepsis of the bench and glassworks with alcohol 70%. Therefore, it was necessary to use all the procedures to avoid possible contamination of the product.

### Treatment

After seven days of stoma creation, infusions of the rectal enemas were started, with daily frequency, always at the same time, for 21 days, with a syringe connected to the urethral probe 6F, according to the group, ie, control group subjected to saline solution injection and Curcuma long group to the *Curcuma longa* extract injection*.*

### Euthanasia and collection of specimens

Euthanasia was performed after 21 days of treatment in all animals by intraperitoneal injection of a folded dose of the anesthetics already described. Then, a new median laparotomy and resection of 2 cm of the proximal rectum were performed, followed by opening the specimen, washing in saline solution and placing in separate and identified tubes containing 10% formaldehyde for histological analysis.

### Histopathological study

**FIGURE 1:**
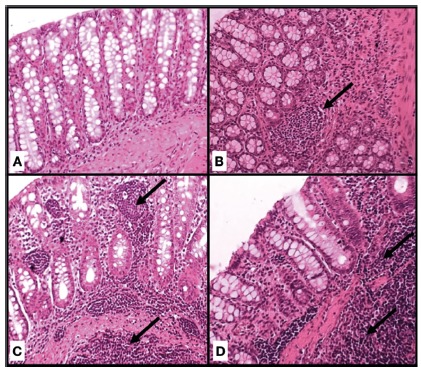
Classification of inflammatory infiltrate by inflammatory focus by field: A) normal epithelium, absence of inflammation; B) one focus; C) two foci; D) more than two foci (H&E 200x)

The specimens were processed, embedded in paraffin and sectioned into a microtome every 5μm; the slides were stained with H&E and evaluated by an experienced researcher without his knowledge of the group. All laminae were analyzed by evaluating the degree of inflammatory process and vascular congestion according to a score varying from 0 to 3. The inflammatory was done based on foci per field (Figure 1) and they were characterized by the presence of macrophages, dendritic cells, B and T lymphocytes observed in the lymphoid follicles exhibiting atrophied mucosa: 0=absence; 1=mild - one focus; 2=moderate - two foci; 3=intense - three foci. Each inflammatory focus was characterized by the presence of mononuclear cell infiltrates including macrophages, lymphocytes and plasma cells, resulting in continuous recruitment of blood circulation and local proliferation. 

### Statistical analysis

The means of the degrees of inflammation and vascular congestion were submitted to statistical treatment by the BioEstat 5.3 program, with analysis of variance (ANOVA), Kruskal-Wallis and Mann-Whitney tests followed by the Tukey test with a significance level of p<0.05.

**FIGURE 2:**
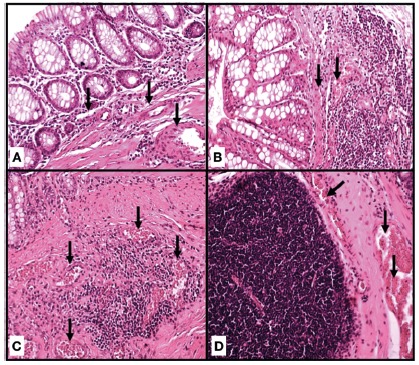
Classification of vascular congestion: A) absence of vascular congestion; B) mild vascular congestion (arrows); C) moderate vascular congestion; D) intense vascular congestion (H&E, 200x)

Classification of vascular congestion (Figure 2): 0=absent; 1=mild; 2=moderate; 3=intense. The criteria for determining the intensity of vascular congestion were pronounced dilation and engorgement of the arterioles and capillaries, with leukocytes in a peripheral position in the laminar flow.

## RESULTS

In the evaluation of the inflammatory process, we observed a mean of 2.7 in the control group, while in the *Curcuma longa* group the mean was 2.6 (p=0.3125, Table 1).

**TABLE 1:** Evaluation of the inflammatory process in the excluded intestinal segment

Rats	Groups	
	Control	Curcuma longa
1	3	0
2	3	3
3	2	2
4	3	3
5	3	3
6	3	3
7	3	3
8	2	3
9	-	3
10	-	3
Mean	2.7	2.6

In relation to the degree of vascular congestion there was no difference between the groups, since in the control group thee observed mean was 2.3, while in the *Curcuma longa* group it was 2.1 (p=0.1642, Table 2). 

**TABLE 2:** Evaluation of the degree of vascular congestion in the excluded intestinal segment

Rats	Groups	
	Control	Curcuma longa
1	0	1
2	1	2
3	3	2
4	3	2
5	3	2
6	2	2
7	3	2
8	3	3
9	-	3
10	-	2
Mean	2.3	2.1

## DISCUSSION

In the present study, it was observed that the exclusion of faecal transit in the colonic segment in rats for 21 days actually promoted an inflammatory process, considering the average of 2.7 degrees observed, on a scale ranging from 0 to 3, characterized by mild to severe inflammation. The same has been observed by other authors, and this is the starting point for the evaluation of the therapeutic efficacy of *Curcuma longa* ^1^ ^,^ ^2^ ^,^ ^3^ ^,^ ^4^ ^,^ ^5^. The method of histological evaluation was also the same, which facilitates comparison, as it is well characterized by infiltration with mononuclear cells, which include macrophages, lymphocytes and plasma cells; tissue destruction by inflammatory cells and attempts at healing by the replacement of tissue damaged by connective tissue by small vessel proliferation (angiogenesis) and fibrosis^7^ ^,^ ^8^ ^,^ ^13^ ^,^ ^14^ ^,^ ^15^ ^,^ ^16^.

Kadri et al.^8^ observed the efficacy of *Curcuma longa* extract in experimental model of exclusion colitis in rats, in contrast to the observed here. This discrepancy in the results may be due to the vehicle used, since in the present research aqueous extract was used, while the authors used oily extract, which in theory could offer greater adhesion of the product to the rectal mucosa. However it seems to us conflicting that those authors did not observe a difference between 50 mg/kg/day and 200 mg/kg/day, that is, even with a concentration four times lower than the one used here also obtained reduction of the inflammatory process in the excluded segment. Kadri et al.^8^ also used histological analysis as applied here, but also analyzed tissue concentration of myeloperoxidase. Another important difference observed in the method was that in the present research we chose to wait seven days to start the treatment, since in clinical practice, exclusion colitis is only treated once it is installed. Kadri et al.^8^ opted to start therapy immediately after the stoma preparation, which would allow a lower degree of inflammation compared to our research.

Fernandez et at.^4^ evaluated the sucralfate in the treatment of exclusion colitis in rats and also started the therapy immediately after the stoma creation, which supports the method of Kadri et al.^8^, but which in our view would not be ideal, because the actual efficacy of the drug is not known in the face of a more intense and already installed inflammatory process. We believe that experimental research should simulate clinical situations so that there is applicability.

Although widely studied as an anti-inflammatory agent, specifically in exclusion colitis, there is only the publication of Kadri et al^8^ using *Curcuma longa*. Thus, it is up to us to compare the results obtained here with other situations of intestinal inflammation. Cunha Neto et al.^3^ observed in a review article on the effects of *Curcuma longa* on the inflammatory bowel process in diseases such as ulcerative colitis and Crohn’s disease; this plant could act as a coadjuvant, since it is proven to reduce oxidative stress and inhibit leukocyte migration. It can also prevent apoptosis of intestinal cells and induce mucosal recovery. Although such effects described are the same as those desired in exclusion colitis to demonstrate the reduction of the inflammatory process, it must be considered that the review analyzed articles with very different methods and none of them in experimentally exclusion colitis.

Simadibrata et al.^16^ also reviewed the effect of *Curcuma longa* on maintaining ulcerative colitis in remission. They found 49 publications on the subject, all in patients with the disease treated by traditional agents and, although the promising result, the authors themselves emphasize the importance of research designed specifically for the pure analysis of *Curcuma longa* and a larger number of patients, since the publications studied had a small number of patients. This publication clearly shows the growing interest in the use of herbal medicines, including *Curcuma longa* in the treatment of colitis.

Vascular congestion is a common finding in the intestinal inflammatory process regardless of origin. It is also one of the earliest, occurring even in mild inflammation^1^ ^,^ ^6^ ^,^ ^10^ ^,^ ^12^. Thus, the fact that there was no statistically significant difference between the groups studied regarding vascular congestion was actually expected, since no difference was also observed in relation to inflammation. 

The results obtained here, although divergent from most publications related to *Curcuma longa* in preventing or reducing the inflammatory bowel process, should not be understood as a negative evaluation of this herbal medicine, since the method used here is different from the publications used for comparison. They only show that when the inflammation is already installed after one week of stoma preparation, therefore with moderate to intense inflammatory process, *Curcuma longa* extract was not effective in decreasing the intensity of the inflammation with the treatment performed for three weeks. It would be advisable to carry out the treatment from the moment the stoma was made in order to obtain a direct comparison, or, what would be ideal in our view, to prolong the treatment for another one to two weeks, which should be the object of further research. 

## CONCLUSION

The extract of *Curcuma longa* was not able to minimize the inflammatory process of the intestinal segment excluded from rats subjected to terminal colostomy.
